# A Necroptosis-Related Prognostic Model of Uveal Melanoma Was Constructed by Single-Cell Sequencing Analysis and Weighted Co-Expression Network Analysis Based on Public Databases

**DOI:** 10.3389/fimmu.2022.847624

**Published:** 2022-02-15

**Authors:** Jiaheng Xie, Liang Chen, Qikai Tang, Wei Wei, Yuan Cao, Chuyan Wu, Jing Hang, Kai Zhang, Jingping Shi, Ming Wang

**Affiliations:** ^1^ Department of Burn and Plastic Surgery, The First Affiliated Hospital of Nanjing Medical University, Jiangsu Province Hospital, Nanjing, China; ^2^ Department of General Surgery, Fuyang Hospital Affiliated to Anhui Medical University, Fuyang, China; ^3^ Department of Neurosurgery, The First Affiliated Hospital of Nanjing Medical University, Jiangsu Province Hospital, Nanjing, China; ^4^ Fourth School of Clinical Medicine, Nanjing Medical University, Nanjing, China; ^5^ Rehabilitation Center, The First Affiliated Hospital of Nanjing Medical University, Nanjing, China; ^6^ Department of Ultrasound in Medicine, The First Affiliated Hospital of Nanjing Medical University, Jiangsu Province Hospital, Nanjing, China; ^7^ Pancreas Center, The First Affiliated Hospital With Nanjing Medical University, Nanjing, China

**Keywords:** uveal melanoma, necroptosis, prognostic model, bioinformatics, ITPA

## Abstract

**Background:**

Uveal melanoma(UVM) is the most common intraocular malignancy and has a poor prognosis. The clinical significance of necroptosis(NCPS) in UVM is unclear.

**Methods:**

We first identified necroptosis genes in UVM by single-cell analysis of the GSE139829 dataset from the GEO database and weighted co-expression network analysis of TCGA data. COX regression and Lasso regression were used to construct the prognostic model. Then survival analysis, immune microenvironment analysis, and mutation analysis were carried out. Finally, cell experiments were performed to verify the role of ITPA in UVM.

**Result:**

By necroptosis-related prognostic model, UVM patients in both TCGA and GEO cohorts could be classified as high-NCPS and low-NCPS groups, with significant differences in survival time between the two groups (P<0.001). Besides, the high-NCPS group had higher levels of immune checkpoint-related gene expression, suggesting that they might be more likely to benefit from immunotherapy. The cell experiments confirmed the role of ITPA, the most significant gene in the model, in UVM. After ITPA was knocked down, the activity, proliferation, and invasion ability of the MuM-2B cell line were significantly reduced.

**Conclusion:**

Our study can provide a reference for the diagnosis and treatment of patients with UVM.

## Introduction

Although uveal melanoma (UVM) is a rare tumor type, it is the most common intraocular malignancy (1). UVM originates in the uveal tract and includes the iris, ciliary body, and retinal choroid (2). It is worth mentioning that UVM and cutaneous malignant melanoma (the most common type of melanoma) have significant differences in risk factors, pathological features, and treatment outcomes (3, 4). However, the current treatment options for UVM are still limited, especially for the treatment of advanced UVM, which is still largely based on the treatment options for cutaneous malignant melanoma (5). Although early detection of UVM has made fairly rapid progress, almost 50% of patients develop metastases resulting in a poor prognosis (6). The most common sites of UVM metastasis are the liver, lung, soft tissue, and bone (7). Once metastasis occurs, there is no effective treatment option, and most patients with metastatic UVM survive less than 12 months (8). Immune checkpoint inhibitor therapies, such as PD-1/PDL-1 or CTLA-4 inhibitors, that have made breakthroughs in the treatment of other solid tumors with a high mutation load, are being investigated for their utility in UVM (9). However, at present, a large number of UVM patients do not respond well to immune checkpoint inhibitors, which may be related to low mutation load, new immune checkpoint acquisition, and the production of immunosuppressive factors (10). Therefore, it is necessary to explore novel biomarkers of UVM and understand their significance in the tumor microenvironment.

Resistance to apoptosis has been recognized as one of the most common hallmarks of cancer, troubling the development of cancer biology (11). Overactivation of growth signals, metabolic reprogramming, and changes in the immune microenvironment are all involved in the process of cancer cell resistance to death, allowing it to proliferate unchecked (12). Inducing the death of cancer cells has become a promising development direction in the field of cancer therapy (12). Currently, the mainstream programmed cell deaths include apoptosis, pyroptosis, necroptosis, ferroptosis, all of which play important roles in homeostasis regulation, inflammatory response, anti-infection, and carcinogenesis (13–15). Necroptosis used to be considered as the “accidental death” of cells, but with further research, the mechanism of necroptosis has been preliminarily explained, and there are obvious differences between necroptosis and traditional cell necrosis (16). Necroptosis is a programmed cell death pathway that is dependent on the caspase system and different from apoptosis (16). The tumor necrosis factor (TNF) family is considered to be the primary activator of necroptosis, by binding to TNFR1 on the plasma membrane, causing the TNF receptor-associated death domain (TRADD) to signal RIPK1 to recruit RIPK3 to form necrosome (17). It’s worth noting that caspase-8, a key molecule in apoptosis, is a necroptosis inhibitor (16). When caspase-8 is activated, RIPK activity is blocked and apoptosis occurs. When caspase-8 is inactivated, RIPK1 and RIPK3 are activated, leading to a series of phosphorylation pathways, which are amplified through cascade reactions. During this process, cell membranes break down and the contents flow out, triggering an immune response (18). Since necroptosis is a surrogate process for apoptosis, inducing necroptosis to kill tumor cells becomes attractive when apoptosis is blocked (19). Furthermore, necroptosis, as one of the immunogenic cell death, also plays an important role in the immune microenvironment (20). Changes in the immune microenvironment induced by necrosis are also important in the context of the rising tide of immune checkpoint therapy (21). Hence, it is significant to explore the role of necroptosis in UVM.

Herein, we downloaded the data of UVM patients from TCGA database and downloaded two datasets of GSE139829 and GSE84976 from GEO database. TCGA database is one of the most authoritative and complete databases available. Therefore, we downloaded TCGA’s UVM data for analysis. GSE139829 is the single-cell sequencing data set of UVM and its sample size is relatively large and contains clinical data, so it was selected for single-cell sequencing analysis. GSE84976 is a chip dataset of UVM that contains detailed survival data, so it was selected to validate our analysis results. Through a comprehensive bioinformatics analysis, we constructed the necroptosis-related prognostic model in UVM and divided UVM patients into the high-risk group and low-risk group based on the risk scores. The outcomes of the two groups were significantly different. In addition, we also explored the guiding value of the signature in the immune microenvironment and tumor mutation load. Finally, we verified the function of the most significant gene ITPA in this signature using cell experiments. Our study can provide a reference for the diagnosis and treatment of UVM.

## Methods

### Transcriptome Data Download and Processing

The “TCGAbiolinks” R package was used to download TCGA data as the training cohort. The FPKM data type of the UVM was converted to the TPM data type. Subsequently, 77 transcriptome data samples with complete clinical data were obtained by eliminating non-primary tumor samples. Through the GEO database, UVM dataset GSE84976 was downloaded as the validation cohort. All data were converted by log2 for subsequent analysis.

### Single Cell Sequencing Data Download and Processing

The single-cell data set GSE139829 of UVM was downloaded from the GEO database. The dataset contains a total of 11 samples. Next, we carry out data quality control. We retained cells with less than 10 percent mitochondrial genes, cells with a total number of genes greater than 200, and genes whose expression ranged from 200 to 7000 and were expressed in at least three cells. The number of highly variable genes was set at 3000. These 11 samples were integrated through SCT correction. Then, by setting the “DIMS” parameter as 20, the tSNE method was used to reduce the dimension of data, and by using the “KNN” method, a resolution was set as 1.0 to conduct clustering of cells. Subsequently, cells were annotated by various cell surface markers. Finally, the percentage of necroptosis-related genes in each cell can be obtained by importing necroptosis genes through the “PercentageFeatureSet” function.

### The Acquisition of Necroptosis-Related Genes

In the genecard database, 571 genes related to necroptosis were found. And a total of 68 necroptosis-related genes were identified with a correlation score greater than 1.0.

### Single Sample Gene Set Enrichment Analysis (ssGSEA)

ssGSEA analysis is often used to quantify the specific score of a gene set enriched in a sample. In this study, the scores related to necroptosis in each UVM patient were obtained by ssGSEA analysis.

### Weighted Co-Expression Network Analysis (WGCNA)

WGCNA analysis is a systems biological method used to describe patterns of gene association between different samples. It can be used to identify highly covarying gene sets and to identify candidate biomarker genes or therapeutic targets based on the interconnectedness of each gene set and the association between the gene set and the phenotype. In this study, WGCNA was performed to find the gene modules related to necroptosis score in UVM, and the genes related to necrotic apoptosis were obtained.

### Construction of the Prognostic Model Associated With Necroptosis

Firstly, the genes related to necroptosis with prognostic value were preliminarily obtained by univariate COX analysis. Subsequently, prognostic necroptosis-related genes were further screened by the least absolute and Selection operator (LASSO) regression, and the prognostic model was constructed. In this way, each UVM can be calculated with a NCPS score through the formula. Based on the median value, patients in the TCGA UVM cohort can be divided into high and low risk groups. We then explored the difference in prognosis between the two groups and assessed the accuracy of the model.

### External Validation of the Model

GSE84976 cohort in GEO was selected as an external validation cohort. In the GSE84976 validation cohort, NCPS scores for each sample were calculated according to the formula of the model, and patients were divided into high- and low-risk groups based on the median. Next, survival analysis was performed to determine whether there was also a difference in prognosis between the high- and low-NCPS subgroups in the validation cohort. The ROC curve was used to evaluate the accuracy of the model. Principal component analysis (PCA) was used to explore whether the model could better group UVM patients.

### Correlation Analysis of Immune Infiltration and Mutation

We downloaded the results calculated by 7 immune infiltration assessment algorithms for all patients in the TCGA database from the Timer2.0 database and extracted the data of UVM patients. Subsequently, we explored the differences of immune cell infiltration among different NCPS groups and showed the immune cells at different levels of infiltration in the form of heat maps. At the same time, the immune checkpoint-related genes differentially expressed in different NCPS subgroups were presented in the form of a boxplot. Finally, we explore the analysis of mutations between groups and show the top 20 genes with the highest mutation frequency.

### The Construction of a Nomogram

In this study, a Nomogram was constructed to assess the risk of death in UVM patients by combining clinical data and NCPS values of the model. Finally, the nomogram’s accuracy in estimating patient outcomes was evaluated by prognostic ROC curves.

### Cell Culture and Transfection

Human Uveal Melanoma cells were purchased from the Fuheng Biology Inc. (Fuheng, Shanghai, China), and were cultured in Roswell Park Memorial Institute 1640 Medium (RPMI 1640) (Gibco) supplemented with 10% FBS (Gibco). Cells were transfected with the previously synthesized small interfering RNAs (GenePharma Inc, Shanghai, China) targeting gene ITPA using the Lipofectamine3000 (Thermo Fisher Scientific, Waltham, MA, USA) according to the manufacturer’s protocol. The siRNA sequences for gene ITPA are provided in **Supplemental Table S1**.

### CCK-8 Assay

The Cell Counting Kit-8 (CCK8) method was used to detect MUM-2B cell viability. The cells were seeded in a 96-well cell culture plate and were subjected to siRNA transfection after adherence. 24 hours after siRNA transfection, CCK-8 solution (Biosharp, Hefei, China) was added to each well of the plate. The plate was subsequently incubated in dark conditions in the cell culture incubator. Finally, the absorbance at 450 nm wavelength was detected using a microplate reader (Molecular Device). All data were presented as the means ± SD of three independent experiments. *P<0.05, **P<0.01, ***P<0.001.

### Quantitative Real−Time Polymerase Chain Reaction (qRT-PCR)

We used qRT-PCR to detect the knockdown potency of the synthesized siRNAs. Total cellular RNAs were isolated using Trizol Reagent (Invitrogen, Carlsbad, CA, USA) according to the manufacturer’s instructions. The reverse transcription and qRT-PCR methods were described in our previous work (5). Relative quantification was determined using the -2ΔΔCt method. The relative expression of messenger RNA (mRNA) for each gene was normalized to the level of glyceraldehyde-3-phosphate dehydrogenase (GAPDH) mRNA. The primers were synthesized by Genscript Biotechnology Inc (Nanjing, China), the sequence of which were listed in **Supplementary Table S2**. All data were presented as the means ± SD of three independent experiments. *P<0.05, **P<0.01, ***P<0.001.

### Scratch Wound Healing Assay

Scratch wound healing assay was used to assess the migration of MuM2B cells. The methods were described in our previous works (5). All data were presented as the means ± SD of three independent experiments. *P<0.05, **P<0.01, ***P<0.001.

### Transwell Assay

Upon transfection, cells were seeded into the upper well for 14h and allowed to invade through the transwell plate. The upper chamber of the plate was precoated with or without Matrigel solution (BD Biosciences), to assess the invasion and migration capacity of cells separately. The cells on the inserts were fixed with methanol, stained with crystal violet, and counted under a light microscope. The percentage of cells successfully migrated through the transwell plate was subjected to statistical analysis. All data were presented as the means ± SD of three independent experiments. *P<0.05, **P<0.01, ***P<0.001.

### Clone Formation Assay

Clone Formation Assay was used to assess the proliferation capacity of MuM2Bs. The MuM-2B cells were seeded on a 6 well plate before siRNAs transfection. After transfection, the cells were incubated in a cell culture incubator for 10 days. The cells were then rinsed with PBS, fixed with methanol, stained with crystal violet, and eventually photographed using a digital camera (Canon). All data were presented as the means ± SD of three independent experiments. *P<0.05, **P<0.01, ***P<0.001.

## Results

**Figure 1:**
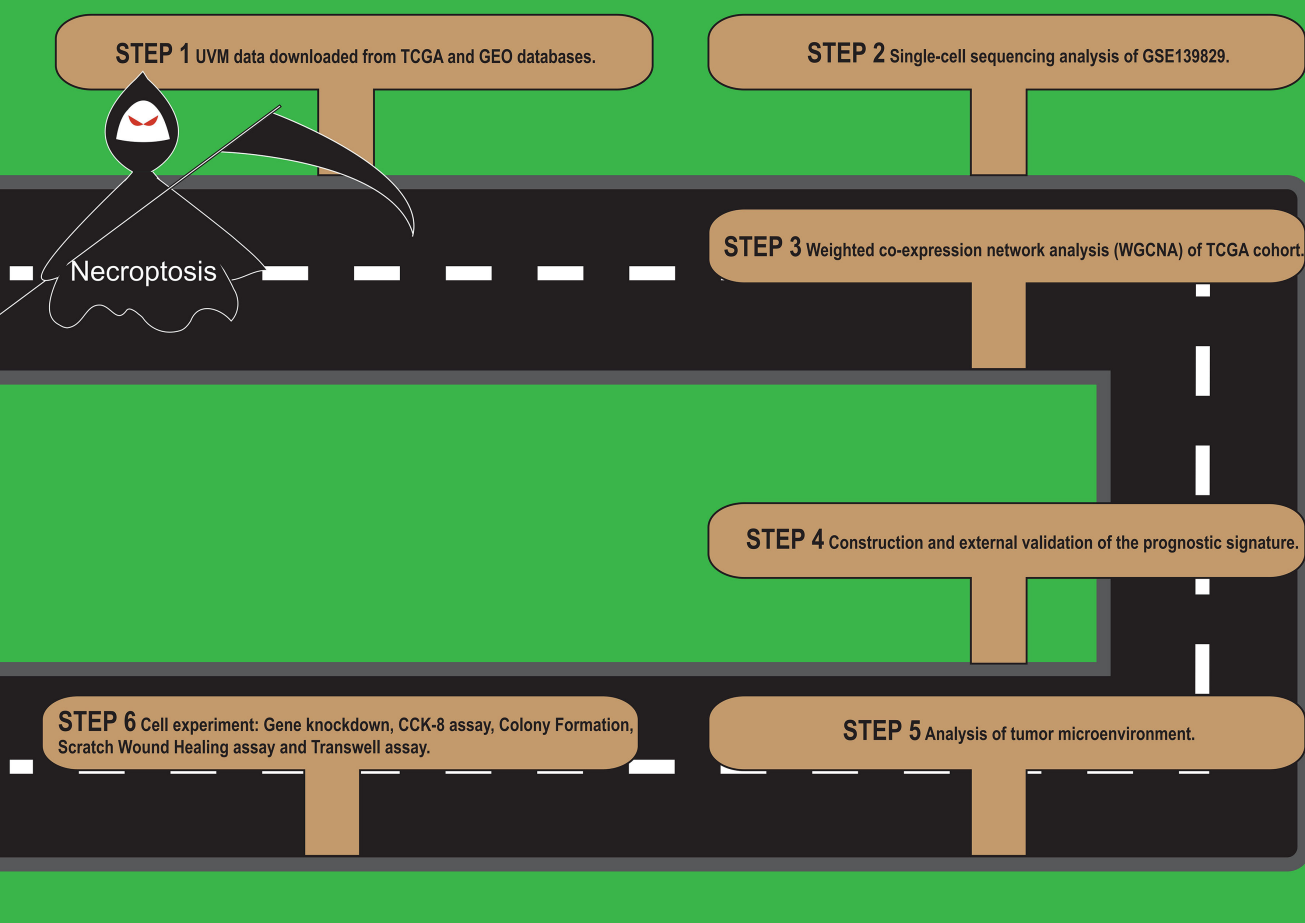
Flow chart.

A flow chart of our work was shown in **Figure 1**.

### Single Cell Sequencing Data Analysis

**Figure 2:**
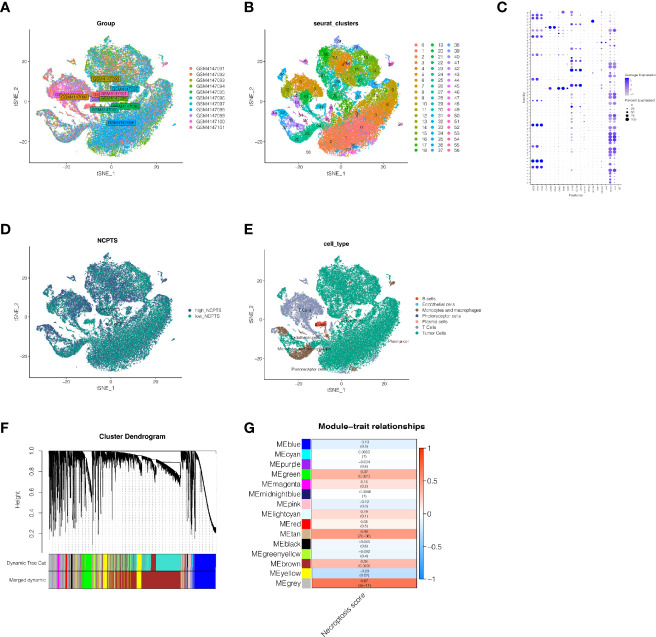
Single cell sequencing analysis of GSE139829. **(A)** The integration effect of 11 samples is good. **(B, C)** Dimensionality reduction and cluster analysis. All cells in 11 samples were clustered into 57 clusters. **(D)** The percentage of necroptosis genes in each cell. The cells were divided into high- and low-necroptosis cells. **(E)** According to the surface marker genes of different cell types, the cells are annotated as B cells, endothelial cells, monocyte and macrophages, photoreceptor cells, plasma cells, T cells and Tumor cells, respectively. **(F, G)** WGCNA found that MEgreen, MEtan, and MEbrown modules were closely related to the score of necroptosis.

We first analyzed the single-cell sequencing dataset GSE139829 of UVM to integrate different samples. As shown in **Figure 2A**, the integration effect among 11 samples was good without an obvious batch effect, which could be used for subsequent analysis. Then we clustered all the cells into 57 clusters by k-Nearest Neighbor (KNN) clustering algorithm (**Figures 2B, C**). Then 68 genes related to necroptosis were input using the “PercentageFeatureSet” function, and finally, the percentage of necroptosis genes in each cell was obtained. The cells were divided into low and high necroptosis cells by their median necroptosis gene proportion and were displayed by tSNE diagrams (**Figure 2D**). Then, according to the surface marker genes of different cell types (**Supplementary Table S3**), we observed their expression in different clusters (**Figure 2C**) and finally determined 7 cell types. B cells, endothelial cells, monocyte and macrophages, photoreceptor cells, plasma cells, T cells, and Tumor cells, respectively (**Figure 2E**). We then analyzed the differentially expressed genes between the high-necroptosis and low-necroptosis groups and 3,673 genes were identified

### Weighted Co-Expression Network Analysis

In the TCGA cohort, gene modules associated with the necroptosis phenotype were obtained by WGCNA analysis of 77 samples. By setting the soft threshold as 9, the minimum number of module genes as 100, deepSplit as 2, and merging the modules with similarity lower than 0.5, a total of 14 non-gray modules were obtained (**Figure 2F**). We found that, as shown in **Figure 2G**, MEgreen, MEtan, MEbrown were closely related to the score of necroptosis in the non-gray module. Genes with a threshold of the p-value <0.0001 were selected from these three modules for subsequent analysis.

### Construction and Validation of Necroptosis-Related Prognostic Model

**Figure 3:**
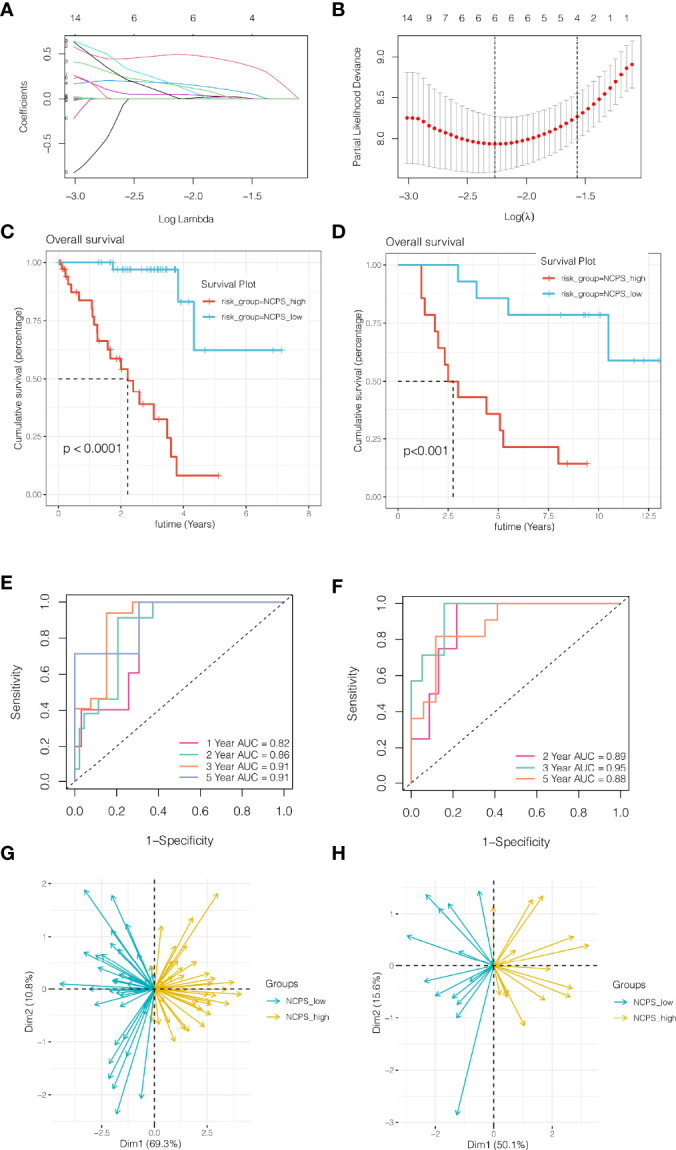
Construction and validation of necroptosis-related prognostic model. **(A, B)** Six genes were selected to construct the prognostic model by Lasso regression. **(C)** Survival analysis of TCGA cohort. The prognosis was significantly worse in the high-NCPS group (P<0.0001). **(D)** Survival analysis of GSE84976 Cohort. The prognosis was significantly worse in the high-NCPS group (P<0.001). **(E)** ROC curve of TCGA cohort. The AUC values of the model in 1, 2, 3 and 5 years were 0.82, 0.86, 0.91 and 0.91, respectively. **(F)** ROC curve of GSE84976 Cohort. The AUC values of the model in 2, 3 and 5 years were 0.89, 0.95 and 0.88, respectively. **(G, H)** PCA analysis in TCGA cohort and GSE84976 cohort. It was found that the model could group UVM patients well in both the training cohort and the validation cohort.

First, we collected 773 genes from the intersection of differentially expressed genes obtained from single-cell sequencing data analysis and genes related to necroptosis obtained from WGCNA. By matching TCGA and GSE84976 coexisting genes, there were 692 genes, which were used for subsequent analysis. In the TCGA cohort, 447 genes related to the prognosis of patients were preliminarily obtained by univariate COX analysis with P <0.05. Then, the random seed was set as 55555 in LASSO regression analysis, and the results showed that when the number of included genes was 6, the gene contraction tended to be stable, and the partial likelihood deviance was minimum, and the optimal LAMDA was 0.104(**Figures 3A, B**). The Lasso regression results of these 6 genes are summarized in **Table 1**. Finally, 6 genes KDELR3, IDH2, S100A6, ITPA, PARP8, and APRC1B were obtained and used to construct the prognostic model. The prognostic model was calculated as follows: NCPS=KDELR3*0.48277442+IDH2*0.17659443+S100A6*0.18023750+ITPA*0.20786934+PARP8*0.05236899+ARPC18*0.07652656. Median values were used to classify patients into high-risk and low-risk groups. In **Figure 3C**, we found that in the TCGA training cohort, the high NCPS group had a poor prognosis (P <0.0001). Similarly, in the GSE84976 validation cohort, we also observed that patients with high-NCPS had a significantly worse prognosis than those with low-NCPS (P <0.001, **Figure 3D**). In order to further explore the accuracy of NCPS in the evaluation of the prognosis of UVM patients, we conducted ROC curve analysis in both the training cohort and the validation cohort. As shown in **Figure 3E**, in the TCGA cohort, the area under the curve (AUC) values at 1, 2, 3 and 5 years were 0.82, 0.86, 0.91, and 0.91, respectively. In the validation cohort, due to the small number of patients with 1-year follow-up, we plotted the ROC curve of prognosis at 2, 3, and 5 years. We found that the areas under the curve at 2,3 and 5 years were 0.89, 0.95, and 0.88, respectively (**Figure 3F**). This indicates that NCPS has high accuracy in predicting patient outcomes in both two cohorts. Finally, PCA analysis was performed on 6 genes in the model in the training set and validation set respectively, and it was found that the model could group UVM patients well in both the training cohort and the validation cohort (**Figures 3G, H** **)**.

**Table 1:** Six genes were identified by Lasso regression to construct a prognostic model.

Gene	HR	P-value
ITPA	12.11	<0.001
ARPC1B	12.107	<0.001
IDH2	4.791	<0.001
KDELR3	4.342	<0.001
S100A6	3.482	<0.001
PARP8	2.602	<0.001

### Immune Infiltration Analysis and Mutation Landscape

**Figure 4:**
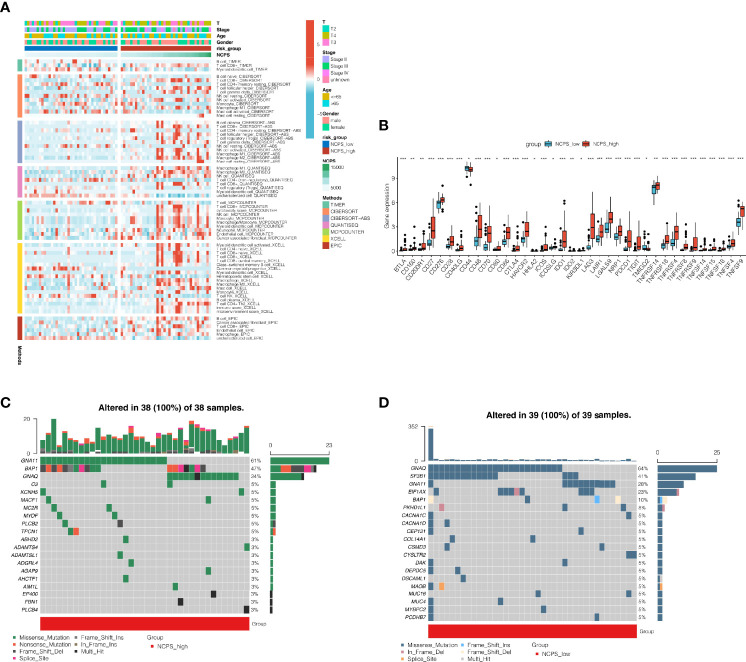
Immune infiltration analysis and mutantion landscape. **(A)** Heat map of immune cell infiltration in high NCPS group and low NCPS group. **(B)** Expression of immune checkpoint related genes in high -NCPS group and low -NCPS group. The results showed that the expression trend of immune checkpoint related genes was higher in the high-NCPS group. **(C)** Mutation landscape of TCGA cohort. **(D)** Mutation landscape of GSE84976 cohort. *P < 0.05; **P < 0.01; ***P < 0.001.

As shown in the above analysis, patient outcomes were significantly different between NCPS subgroups. In order to explore the causes and provide a reference for immunotherapy, we explored the differences in immune infiltration levels among different groups. The results showed that, as shown in **Figure 4A**, there were more immune cell infiltrates in the high NCPS group, including macrophage M2 and T cells. Next, the expression of genes associated with immune checkpoints was investigated. As shown in **Figure 4B**, most of the immune checkpoint-related genes, such as PDCD1 and CTLA4, were found to have higher expression levels in the high NCPS group. We hypothesized that the high NCPS group was associated with a higher degree of immune infiltration, but the high expression of immune checkpoint genes may lead to a low response state, and patients in the high NCPS group may benefit more from immune checkpoint inhibitors. Subsequently, we analyzed the mutation landscape of the top 20 mutated genes in the high and low NCPS groups respectively. The results showed that the mutation incidence of the top 20 mutated genes in both two groups was 100%. The most significantly mutated gene in the high NCPS group was GNA11, and the most significantly mutated gene in the low GNAQ group was GNAQ(**Figures 4C, D**).

### Cell Localization of 6 Modeling Genes

**Figure 5:**
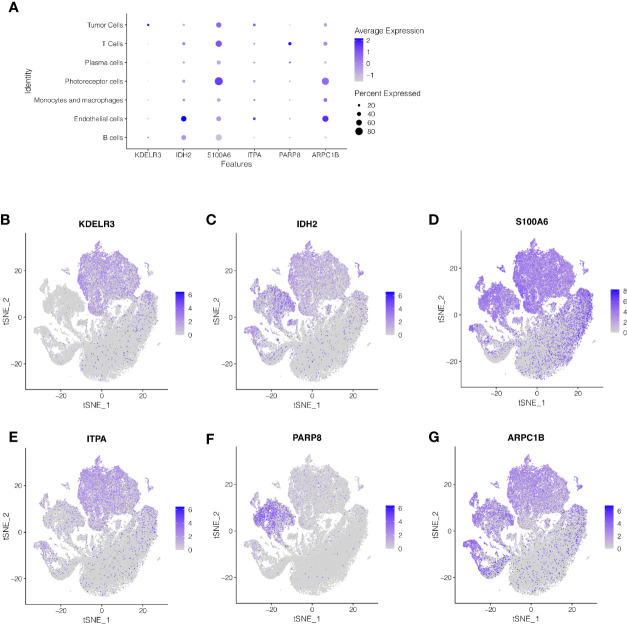
Single-cell sequencing analysis to explore the cell localization of 6 modeling genes. **(A–G)** KDELR3 was mainly expressed in tumor cells, IDH2 was mainly expressed in endothelial cells, S100A6 was mainly expressed in photoreceptor cells, ITPA was mainly expressed in endothelial cells and tumor cells, PARP8 was mainly expressed in T cells, and APRC1B was mainly expressed in endothelial cells.

We performed single-cell sequencing analysis to explore the expression of modeling genes in different cell types. As shown in **Figures 5A–G**, KDELR3 was mainly expressed in tumor cells, IDH2 was mainly expressed in endothelial cells, S100A6 was mainly expressed in photoreceptor cells, ITPA was mainly expressed in endothelial cells and tumor cells, PARP8 was mainly expressed in T cells, and APRC1B was mainly expressed in endothelial cells.

### The Construction of a Nomogram

**Figure 6:**
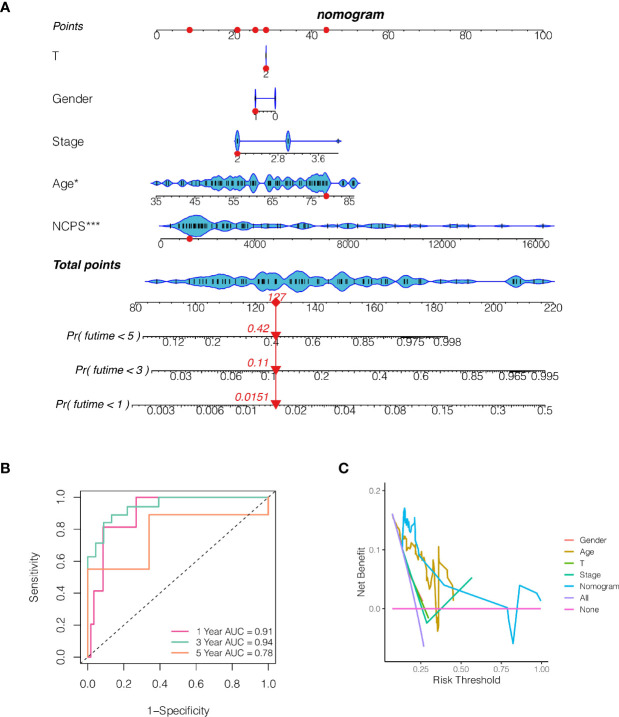
The construction of a nomogram. **(A)** Nomogram of patient “TCGA-VD-AA8R”. The mortality rate of the patient in 1, 3 and 5 years was estimated to be 0.015, 0.11 and 0.42. **(B)** ROC curve of the nomogram. The area under the curve (AUC) in 1, 3 and 5 years were 0.91, 0.94 and 0.78 respectively. **(C)** Decision curve analysis. The effect of this nomogram was superior to other clinical indicators.

To better assess the risk of UVM patients, a nomogram was constructed combining clinical data and NCPS values. As shown in **Figure 6A**, according to the gender, age, T stage, total stage, and NCPS score of the patient “TCGA-VD-AA8R”, the mortality rate of the patient in 1, 3 and 5 years was estimated to be 0.015, 0.11, and 0.42. The nomogram can better assess patient risk and guide subsequent clinical decisions. To further evaluate the accuracy of this nomogram, prognostic ROC analysis was performed. The results showed that the area under the curve (AUC) in 1, 3, and 5 years were 0.91, 0.94, and 0.78 respectively (**Figure 6B**). We also performed the decision curve analysis, which evaluates the clinical decision value by calculating the area of each clinical feature and None’s horizontal axis. Results showed that the effect of this nomogram was superior to other clinical indicators, suggesting that this nomogram has a good effect in predicting the prognosis of patients and can guide clinical decision-making (**Figure 6C**).

### Survival Analysis of ITPA

**Figure 7:**
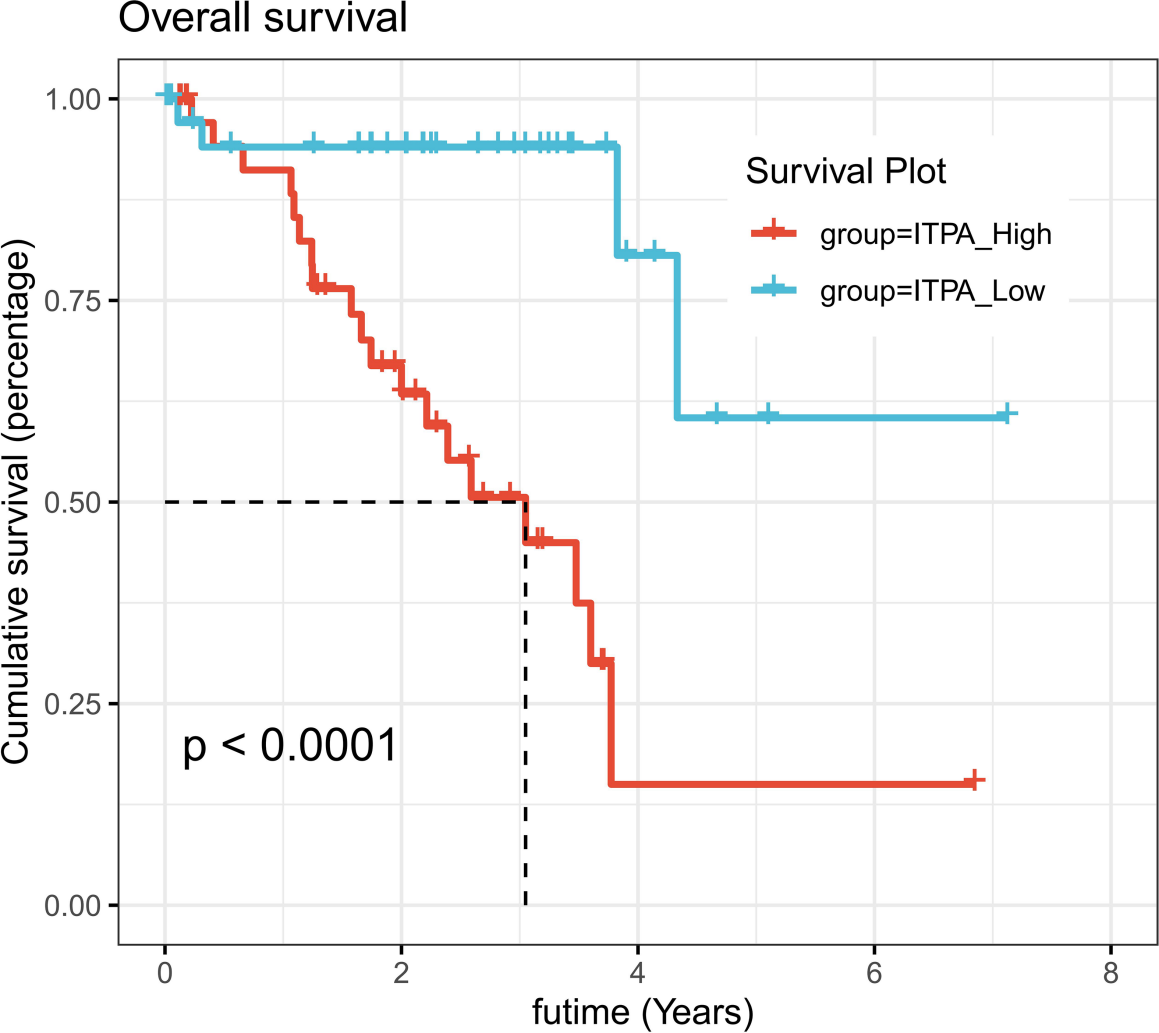
Survival analysis of ITPA in UVM. UVM patients with high expression of ITPA had significantly worse prognosis than patients with low expression of ITPA (P < 0.001).

In COX regression and Lasso regression analysis, The HR value of ITPA was the highest, so we further conducted survival analysis on ITPA. Results showed that patients with high expression of ITPA had a significantly worse prognosis than patients with low expression of ITPA (P<0.001, **Figure 7**).

### ITPA Knockdown Resulted in Reduced MuM-2B Cell Vitality *In Vitro*

**Figure 8:**
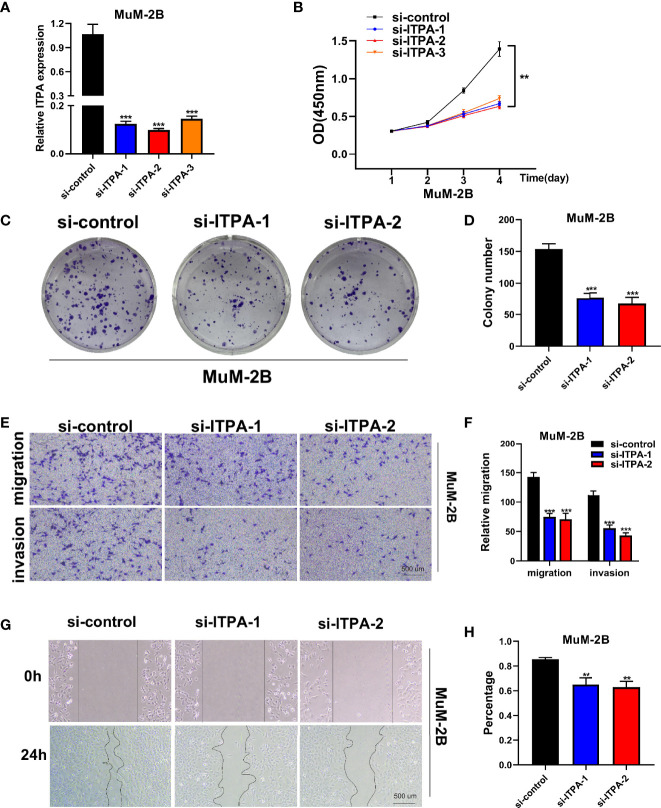
Cell experiment. **(A)** qRT-PCR to evaluate the level of ITPA mRNA 3 days after transfection. All siRNA sequences could result in significant decrease in ITPA mRNA expression (P<0.001). **(B)** CCK8 assay. After ITPA knockdown, the cells showed significant reduction in viability, and si-ITPA-1 and si-ITPA-2 had a better knockdown potency, which were used in further *in vitro* experiments. **(C, D)** Colony formation assay. Cells with a reduced ITPA expression exhibited a significant decrease in the numbers of colonies, compared with the siRNA negative control (NC) group. **(E, F)** Transwell assay. The migration and invasion capacity of MuM-2B cells decreased significantly after ITPA knockdown. **(G, H)** Scratch-wound healing assay. A significantly slower wound healing rate was observed in cells with a decreased expression of ITPA gene. All data were presented as the means ± SD of three independent experiments. *P < 0.05, **P < 0.01, ***P < 0.001.

To assess the capacity of siRNA knockdown of gene ITPA in MuM-2B cell lines, we used the q RT-PCR method to evaluate the level of ITPA mRNA 3 days after transfection (**Figure 8A**). We found that all siRNA sequences could result in a significant decrease in ITPA mRNA expression (P<0.001). CCK8 assay further showed that after ITPA knockdown, the cells showed a significant reduction in viability, and si-ITPA-1 and si-ITPA-2 had a better knockdown potency, which was used further *in vitro* experiments. The experiment results illustrate that the ITPA gene may play a significant role in uveal melanoma cell survival (**Figure 8B**).

### ITPA Plays a Crucial Role in UVM Cell Line Proliferation *In Vitro*

Colony formation assay was used in the assessment of cell proliferation. Cells with a reduced ITPA expression exhibited a significant decrease in the numbers of colonies, compared with the siRNA negative control (NC) group (**Figures 8C, D**). Therefore, a reduced tempo of colony formation was seen in ITPA knockdown cells, indicating that ITPA may play a potentially crucial role in UVM cell line proliferation.

### ITPA Plays a Crucial Role in UVM Cell Line Migration and Invasion *In Vitro*

The migration and invasion capacity of MuM-2B cells also decreased significantly after ITPA knockdown. We found that after siRNA knockdown, the percentage of cells migrated through the transwell plate decreased significantly (**Figures 8E, F**). Scratch-wound healing assay showed a similar result. A significantly slower wound healing rate was observed in cells with a decreased expression of the ITPA gene (**Figures 8G, H**).

## Discussion

Although uveal melanoma and cutaneous melanoma both originate in melanocytes, there are many differences in their biological behaviors and therapeutic responses (22). Known as immunotherapies, such as immune checkpoint inhibitors, have made landmark advances in the treatment of skin melanoma in the 21st century, and thousands of skin melanoma patients have benefited from immunotherapy and extended survival (23). However, immunotherapy has not achieved satisfactory results in UVM (24). In a multicenter retrospective study, the researchers compared the efficacy of conventional chemotherapy with that of immune checkpoint inhibitor-based immunotherapy for UVM and found no significant difference in adjusted OS between immunotherapy patients and chemotherapy patients (25). Mechanisms such as low mutation load and acquisition of new immune checkpoints are thought to be responsible for the low response of UVM to immunotherapy (9). Therefore, exploring the immune microenvironment of UVM becomes attractive. Necroptosis is an inflammatory cell death pathway, and inflammatory molecules released during this process may play an important role in altering the tumor microenvironment (26). The role of Necroptosis in UVM research is unclear. It is time to investigate the prognostic value of Necroptosis in UVM and its role in the immune microenvironment.

In this study, through extensive analysis of UVM data from TCGA and GEO databases, we constructed the prognostic signature of necroptosis-related genes in UVM. By calculating the risk score, patients with UVM could be classified into high-risk and low-risk groups. The high-risk group showed worse outcomes in both TCGA and GEO cohorts (P<0.001). Besides, the ROC curves showed that this signature showed high accuracy in evaluating the prognosis of UVM patients at 1, 3, and 5 years. Analysis of the immune microenvironment showed that high NCPS with high levels of gene expression associated with immune checkpoints may be more likely to benefit from immunotherapy. Subsequent cell experiments showed that the activity, proliferation and invasion ability of UVM were significantly reduced by knocking down ITPA. This suggests that ITPA may be a potential target in UVM.

The potential role of programmed cell death in tumor therapy and the exploration of the immune microenvironment is increasingly emerging, and more and more evidence indicates that targeting pyroptosis, ferroptosis, necroptosis may be a new approach for the next stage of tumor therapy (26). The significance of necroptosis in many tumor types has been preliminarily explained. Xie et al. found that the mTOR/RIPK3/Necroptosis axis is a driving force in inflammatory bowel disease and intestinal cancer (17). Seehawer et al. found that necroptosis-associated microenvironment is involved in hepatocyte transformation into cancer (27). Chefetz et al. found that aldehyde dehydrogenase 1A (ALDH1A) induces necroptosis of cancer cells in ovarian cancer and is involved in the development of chemoresistance (28). However, necroptosis-related studies are lacking in UVM. Our study provides the prognostic signature of necroptosis genes in UVM for the first time, which has significant implications for the prognosis of UVM.

The eyeball is now known for its immune privilege (29). But this immunity privilege is not absolute. A large amount of evidence suggests that both innate and acquired immune processes exist in UVM, and immune privilege may be the mechanism for UVM to escape immune surveillance and promote metastasis (1). Nearly 90% of UVM deaths have liver metastasis, and UVM may have immune privilege mechanism similar to primary eye at the site of liver metastasis, thus forming a relatively suppressed immune microenvironment (30). Understanding the immune microenvironment of UVM is important. Our study found significant differences in levels of immune cell infiltration between the high-risk and low-risk groups. High-NCPS group was associated with high expression of immune checkpoints genes, so high-NCPS group may be more likely to benefit from immunotherapy.

Inosine triphosphate pyrophosphatase (ITPA) has previously been shown to lack the ability to inhibit cell growth and enhance DNA instability in mice (31). In addition, ITPA has been confirmed to be associated with the pathogenesis and drug toxicity of acute lymphoblastic leukemia (32). However, the role of ITPA in UVM has not been discovered. Our study first found that ITPA was the most significant HR value among the six modeled genes, and subsequent survival analysis showed that high expression of ITPA was associated with poor prognosis in UVM patients. Finally, cell experiments confirmed that ITPA gene knockdown in UVM cell lines could significantly inhibit the activity and proliferation and invasion ability of cancer cells. This provides a potential therapeutic target for UVM.

Datasets GSE139829 and GSE84976 we used have preliminarily revealed the changes in the immune microenvironment of UVM, and we conducted in-depth exploration on their basis. In the results that have been published, GSE139829 revealed the heterogeneity of UVM and differences in immune infiltration through single-cell sequencing technology and identified LAG3 as a potential target for immunotherapy (33). GSE84976 was a chip data that reveals the regulatory relationship of HLA molecules in UVM (34). In our study, we first divided UVM cells into two groups with different necroptosis states by further single-cell analysis of GSE139829. This provides a reference for our study of necroptosis heterogeneity in UVM. Subsequently, the prognostic model was constructed based on the differentially expressed genes between the two cell populations. Survival data from the GSE84976 dataset were used to validate the accuracy of the prognostic model. It can be seen that the AUC value of this prognostic model is greater than 0.8, indicating that the model can accurately assess the prognosis of patients with UVM, which is one of the advantages of the model.

To our knowledge, this is the first necroptosis prognostic model of UVM using the single-cell cluster analysis, which not only provides a reference for the study of programmed death in UVM but also helps in the management of patients with UVM.

## Conclusion

We constructed a necroptosis gene prognostic model in UVM. Through this model, we can well evaluate the prognosis and immune microenvironment of UVM patients. We also confirmed the role of ITPA in UVM by cell assay. This provides a potential therapeutic target for UVM.

## Data Availability Statement

The original contributions presented in the study are included in the article/**Supplementary Material**. Further inquiries can be directed to the corresponding authors.

## Author Contributions

JX was responsible for the design of this study. JX, LC, MW, and QT were responsible for all data analysis and manuscript writing. JX, CW, JH, and WW were responsible for writing the manuscript. JS, KZ, and MW provided funding. All the authors agreed on the final version of the article.

## Conflict of Interest

The authors declare that the research was conducted in the absence of any commercial or financial relationships that could be construed as a potential conflict of interest.

## Publisher’s Note

All claims expressed in this article are solely those of the authors and do not necessarily represent those of their affiliated organizations, or those of the publisher, the editors and the reviewers. Any product that may be evaluated in this article, or claim that may be made by its manufacturer, is not guaranteed or endorsed by the publisher.

## References

[1] Chattopadhyay C Kim DW Gombos DS Oba J Qin Y Williams MD. Uveal Melanoma: From Diagnosis to Treatment and the Science in Between. Cancer (2016) 122(15):2299–312. doi: 10.1002/cncr.29727 PMC5567680 26991400

[2] Kaliki S Shields CL. Uveal Melanoma: Relatively Rare But Deadly Cancer. Eye (Lond) (2017) 31(2):241–57. doi: 10.1038/eye.2016.275 PMC5306463 27911450

[3] Kaliki S Shields CL Shields JA. Uveal Melanoma: Estimating Prognosis. Indian J Ophthalmol (2015) 63(2):93–102. doi: 10.4103/0301-4738.154367 25827538 PMC4399142

[4] Xie J Ruan S Zhu Z Wang M Cao Y Ou M. Database Mining Analysis Revealed the Role of the Putative H+/sugar Transporter Solute Carrier Family 45 in Skin Cutaneous Melanoma. Channels (Austin) (2021) 15(1):496–506. doi: 10.1080/19336950.2021.1956226 34334114 PMC8331014

[5] Cao Y Xie J Chen L Hu Y Zhai L Yuan J. Construction and Validation of a Novel Pyroptosis-Related Gene Signature to Predict the Prognosis of Uveal Melanoma. Front Cell Dev Biol (2021) 9:761350. doi: 10.3389/fcell.2021.761350 34901006 PMC8662541

[6] Rusňák Š Hecová L Kasl Z Sobotová M Hauer L. Therapy of Uveal Melanoma A Review. Terapie Uveálního Melanomu Přehled. Cesk Slov Oftalmol (2020) 77(1):1–13. doi: 10.31348/2020/10 33086849

[7] Khan S Carvajal RD. Novel Approaches to the Systemic Management of Uveal Melanoma. Curr Oncol Rep (2020) 22(10):104. doi: 10.1007/s11912-020-00965-0 32725406

[8] Ortega MA Fraile-Martínez O García-Honduvilla N Coca S Álvarez-Mon M Buján J. Update on Uveal Melanoma: Translational Research From Biology to Clinical Practice (Review). Int J Oncol (2020) 57(6):1262–79. doi: 10.3892/ijo.2020.5140 PMC7646582 33173970

[9] Castet F Garcia-Mulero S Sanz-Pamplona R Cuellar A Casanovas O Caminal JM. Uveal Melanoma, Angiogenesis and Immunotherapy, Is There Any Hope? Cancers (Basel) (2019) 11(6):834. doi: 10.3390/cancers11060834 PMC6627065 31212986

[10] Kim DW Anderson J Patel SP. Immunotherapy for Uveal Melanoma. Melanoma Manage (2016) 3(2):125–35. doi: 10.2217/mmt-2015-0006 PMC6094800 30190881

[11] Wong RS. Apoptosis in Cancer: From Pathogenesis to Treatment. J Exp Clin Cancer Res (2011) 30(1):87. doi: 10.1186/1756-9966-30-87 21943236 PMC3197541

[12] Kroemer G Pouyssegur J. Tumor Cell Metabolism: Cancer's Achilles' Heel. Cancer Cell (2008) 13(6):472–82. doi: 10.1016/j.ccr.2008.05.005 18538731

[13] Kim C Kim B. Anti-Cancer Natural Products and Their Bioactive Compounds Inducing ER Stress-Mediated Apoptosis: A Review. Nutrients (2018) 10(8):1021. doi: 10.3390/nu10081021 PMC6115829 30081573

[14] Fang Y Tian S Pan Y Li W Wang Q Tang Y. Pyroptosis: A New Frontier in Cancer. BioMed Pharmacother (2020) 121:109595. doi: 10.1016/j.biopha.2019.109595 31710896

[15] Frank D Vince JE. Pyroptosis Versus Necroptosis: Similarities, Differences, and Crosstalk. Cell Death Differ (2019) 26(1):99–114. doi: 10.1038/s41418-018-0212-6 30341423 PMC6294779

[16] Su Z Yang Z Xu Y Chen Y Yu Q. Apoptosis, Autophagy, Necroptosis, and Cancer Metastasis. Mol Cancer (2015) 14:48. doi: 10.1186/s12943-015-0321-5 25743109 PMC4343053

[17] Xie Y Zhao Y Shi L Li W Chen K Li M. Gut Epithelial TSC1/mTOR Controls RIPK3-Dependent Necroptosis in Intestinal Inflammation and Cancer. J Clin Invest (2020) 130(4):2111–28. doi: 10.1172/JCI133264 PMC7108921 31961824

[18] Wang T Jin Y Yang W Zhang L Jin X Liu X. Necroptosis in Cancer: An Angel or a Demon? Tumour Biol (2017) 39(6):1010428317711539. doi: 10.1177/1010428317711539 28651499

[19] Ogretmen B. Sphingolipid Metabolism in Cancer Signalling and Therapy. Nat Rev Cancer (2018) 18(1):33–50. doi: 10.1038/nrc.2017.96 29147025 PMC5818153

[20] Sprooten J De Wijngaert P Vanmeerbeerk I Martin S Vangheluwe P Schlenner S. Necroptosis in Immuno-Oncology and Cancer Immunotherapy. Cells (2020) 9(8):1823. doi: 10.3390/cells9081823 PMC7464343 32752206

[21] Li X Gong W Wang H Li T Attri KS Lewis RE. O-GlcNAc Transferase Suppresses Inflammation and Necroptosis by Targeting Receptor-Interacting Serine/Threonine-Protein Kinase 3 [Published Correction Appears in Immunity. 2019 Apr 16;50(4):1115]. Immunity (2019) 50(3):576–90.e6. doi: 10.1016/j.immuni.2019.01.007 30995496 PMC6508067

[22] Carvajal RD Schwartz GK Tezel T Marr B Francis JH Nathan PD. Metastatic Disease From Uveal Melanoma: Treatment Options and Future Prospects. Br J Ophthalmol (2017) 101(1):38–44. doi: 10.1136/bjophthalmol-2016-309034 27574175 PMC5256122

[23] Riley RS June CH Langer R Mitchell MJ. Delivery Technologies for Cancer Immunotherapy. Nat Rev Drug Discov (2019) 18(3):175–96. doi: 10.1038/s41573-018-0006-z PMC6410566 30622344

[24] Breazzano MP Milam RW Jr Batson SA Johnson DB Daniels AB. Immunotherapy for Uveal Melanoma. Int Ophthalmol Clin (2017) 57(1):29–39. doi: 10.1097/IIO.0000000000000148 27898611

[25] Rozeman EA Prevoo W Meier MAJ Sikorska K Van TM van de Wiel BA. Phase Ib/II Trial Testing Combined Radiofrequency Ablation and Ipilimumab in Uveal Melanoma (SECIRA-Um). Melanoma Res (2020) 30(3):252–60. doi: 10.1097/CMR.0000000000000653 31895753

[26] Gong Y Fan Z Luo G Yang C Huang Q Fan K. The Role of Necroptosis in Cancer Biology and Therapy. Mol Cancer (2019) 18(1):100. doi: 10.1186/s12943-019-1029-8 31122251 PMC6532150

[27] Seehawer M Heinzmann F D'Artista L Harbig J Roux PF Hoenicke L. Necroptosis Microenvironment Directs Lineage Commitment in Liver Cancer [Published Correction Appears in Nature. 2018 Nov 8]. Nature (2018) 562(7725):69–75. doi: 10.1038/s41586-018-0519-y 30209397 PMC8111790

[28] Chefetz I Grimley E Yang K Hong L Vinogradova EV Suciu R. A Pan-ALDH1A Inhibitor Induces Necroptosis in Ovarian Cancer Stem-Like Cells. Cell Rep (2019) 26(11):3061–75.e6. doi: 10.1016/j.celrep.2019.02.032 30865894 PMC7061440

[29] Keino H Horie S Sugita S. Immune Privilege and Eye-Derived T-Regulatory Cells. J Immunol Res (2018) 2018:1679197. doi: 10.1155/2018/1679197 29888291 PMC5985108

[30] Jager MJ Shields CL Cebulla CM Abdel-Rahman MH Grossniklaus HE Stern MH. Uveal Melanoma. Nat Rev Dis Primers (2020) 6(1):24. doi: 10.1038/s41572-020-0158-0 32273508

[31] Sakumi K Abolhassani N Behmanesh M Iyama T Tsuchimoto D Nakabeppu Y. ITPA Protein, an Enzyme That Eliminates Deaminated Purine Nucleoside Triphosphates in Cells. Mutat Res (2010) 703(1):43–50. doi: 10.1016/j.mrgentox.2010.06.009 20601097

[32] Simone PD Pavlov YI Borgstahl GEO. ITPA (Inosine Triphosphate Pyrophosphatase): From Surveillance of Nucleotide Pools to Human Disease and Pharmacogenetics. Mutat Res (2013) 753(2):131–46. doi: 10.1016/j.mrrev.2013.08.001 PMC3827912 23969025

[33] Durante MA Rodriguez DA Kurtenbach S Kuznetsov JN Sanchez MI Decatur CL. Single-Cell Analysis Reveals New Evolutionary Complexity in Uveal Melanoma. Nat Commun (2020) 11(1):496. doi: 10.1038/s41467-019-14256-1 31980621 PMC6981133

[34] van Essen TH van Pelt SI Bronkhorst IH Versluis M Némati F Laurent C. Upregulation of HLA Expression in Primary Uveal Melanoma by Infiltrating Leukocytes. PloS One (2016) 11(10):e0164292. doi: 10.1371/journal.pone.0164292 27764126 PMC5072555

